# A review of exercise interventions for reducing anxiety symptoms: Insights and implications

**DOI:** 10.1097/MD.0000000000040084

**Published:** 2024-10-11

**Authors:** Zimakor Ewuzie, Chimezirim Ezeano, Nicholas Aderinto

**Affiliations:** a Cygnet Hospital, Harrogate, North Yorkshire, United Kingdom; b University of North Texas, Health Science Center, Fort Worth, TX, USA; c Ladoke Akintola University of Technology, Ogbomoso, Nigeria.

**Keywords:** aerobic exercise, anxiety symptoms, generalized anxiety disorder, non-pharmacological interventions

## Abstract

Generalized anxiety disorder (GAD) is a prevalent mental health condition affecting a significant proportion of the adult population. Despite the availability of pharmacological treatments, their long-term efficacy and potential side effects necessitate exploring alternative interventions. Aerobic exercise has emerged as a promising non-pharmacological approach for managing anxiety symptoms in individuals with GAD. This narrative review examines the efficacy of aerobic exercise interventions in alleviating symptoms of anxiety disorders, drawing on a comprehensive analysis of relevant literature. The review synthesizes findings from studies investigating various forms of aerobic exercise, including high-intensity interval training, resistance training, Pilates, and walking. The results indicate that aerobic exercise interventions demonstrate efficacy in reducing anxiety symptoms and improving overall well-being across diverse populations, including primary care patients, individuals with coronary heart disease, and older adults with cancer undergoing chemotherapy. The review discusses the neurobiological and psychological mechanisms underlying the anxiolytic effects of aerobic exercise. It highlights the implications of these findings for clinical practice, public health initiatives, and future research directions. Despite the promising evidence, limitations in study methodologies and heterogeneity across interventions warrant a cautious interpretation of the results. Further research is needed to elucidate optimal exercise modalities, dosages, and long-term effects on anxiety outcomes.

## 1. Introduction

Generalized anxiety disorder (GAD) stands as among the prevailing mental health conditions, impacting a substantial portion of the adult population, with estimates suggesting up to 20% are affected annually.^[1]^ The prevalence is approximately twice as high among women as among men. Individuals with GAD experience persistent and uncontrollable feelings of apprehension, tension, and nervousness.^[2]^ This chronic anxiety can significantly interfere with their daily functioning.^[3]^ Given the substantial burden of GAD on affected individuals and society as a whole, there is a critical need to explore effective interventions for managing anxiety symptoms in this population.^[4]^ While pharmacological treatments are commonly used, they are associated with side effects and limitations in long-term efficacy.^[5]^ Consequently, there is growing interest in non-pharmacological approaches, such as aerobic exercise, as complementary or alternative treatments for GAD.^[5]^

Aerobic exercise is renowned for its physical health benefits.^[6]^ Perhaps most notable are improvements in cardiovascular fitness and weight management.^[7]^ In addition to its well-documented physical benefits, emerging research suggests that aerobic exercise positively influences mental health.^[8]^ Studies have demonstrated that regular participation in aerobic activities is associated with reduced symptoms of GAD and depression, enhanced mood, and improved psychological well-being.^[9,10]^ From a biological perspective, aerobic exercise stimulates the release of endorphins, neurotransmitters in the brain known for their mood-enhancing properties.^[11]^ These “feel-good” chemicals help alleviate stress, reduce feelings of anxiety and depression, and promote a sense of relaxation and well-being.^[12]^ Furthermore, aerobic exercise has been shown to increase the production of neurotrophic factors, such as brain-derived neurotrophic factor (BDNF), which support the growth and maintenance of neurons in the brain, contributing to improved cognitive function and resilience to stress.^[13]^

Psychologically, regular aerobic exercise is a powerful coping strategy for managing stress and negative emotions.^[14]^ The rhythmic and repetitive nature of aerobic activities can induce a state of mindfulness, diverting attention away from worrisome thoughts and promoting a sense of present-moment awareness and tranquility.^[15]^ Additionally, the sense of accomplishment and self-efficacy accompanying regular exercise can bolster self-esteem and confidence, further enhancing mental well-being.^[16]^ This review examines the current body of research investigating the efficacy of aerobic exercise interventions in reducing anxiety symptoms among adults diagnosed with GAD.

## 2. Methodology

**Figure 1.**
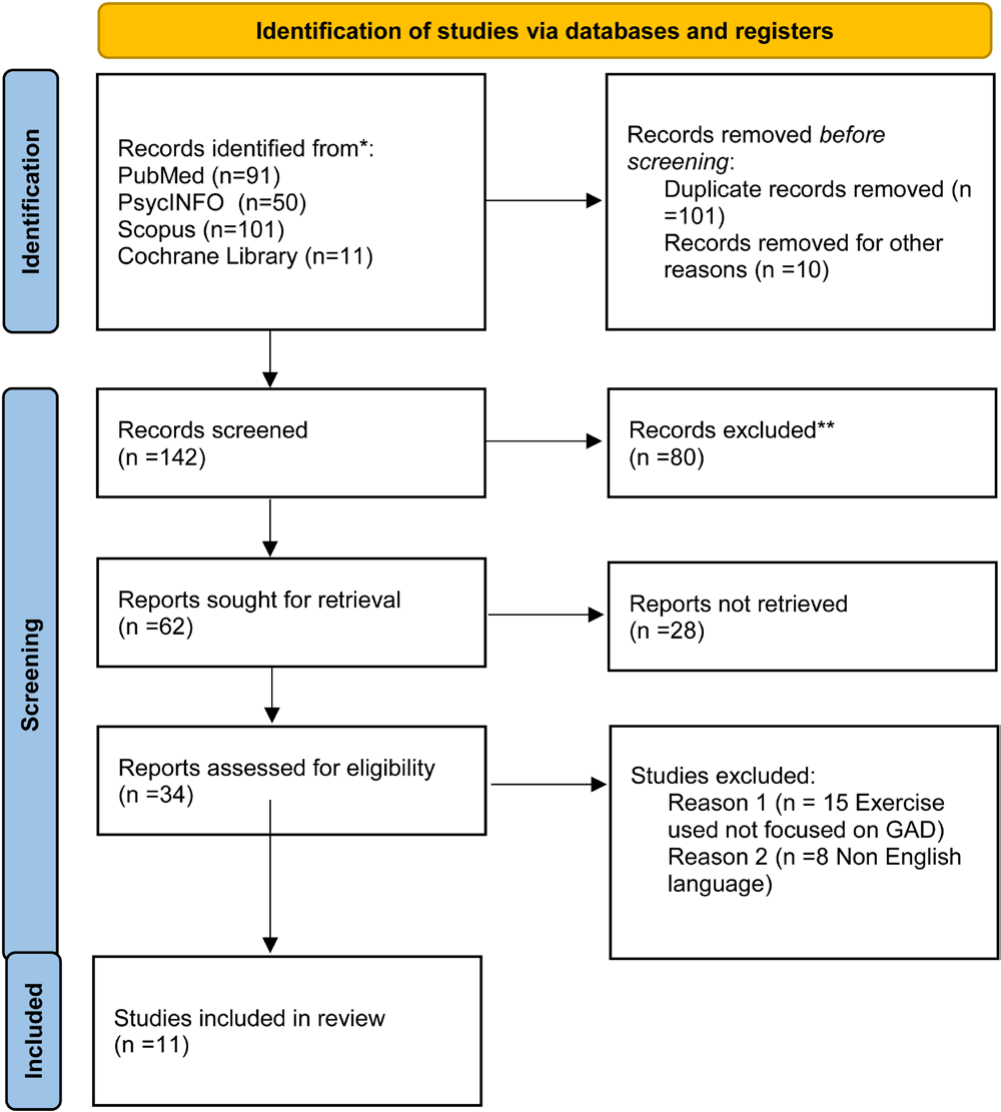
Screening process.

The literature search used PubMed, PsycINFO, SCOPUS and the Cochrane Library. The search strategy used keywords and Medical Subject Headings (MeSH) terms to identify relevant literature (Fig. 1). Key terms included “aerobic exercise,” “anxiety disorder,” “generalized anxiety disorder,” “quality of life,” and variations thereof. MeSH terms ((“exercise”[MeSH Terms] OR “exercise”[All Fields]) AND “interventions”[All Fields] AND Reducing [All Fields] AND (“anxiety”[MeSH Terms] OR “anxiety”[All Fields]) AND (“diagnosis”[Subheading] OR “diagnosis”[All Fields] OR “symptoms”[All Fields] OR “diagnosis”[MeSH Terms])) related to anxiety disorders and exercise were also utilized to enhance the precision and sensitivity of the search.

The review included primary research studies, such as randomized controlled trials (RCTs) and observational studies that investigated the effectiveness of aerobic exercise interventions in reducing anxiety symptoms and improving quality of life among adults with GAD. Studies published in peer-reviewed journals were considered for inclusion. Eligible participants were adults aged 18 to 65 who met diagnostic criteria for GAD according to standardized criteria. Studies not published in English, reviews, conference abstracts were excluded. Search included studies from 2000 to February 2024.

Relevant studies were identified through a screening process. Initially, titles and abstracts were screened based on predefined inclusion and exclusion criteria to identify potentially relevant articles. Full-text articles were then assessed for eligibility, with discrepancies resolved through discussion or consultation with a third reviewer if necessary. Data extraction was performed using a standardized form to capture key information from included studies, including study design, participant characteristics, intervention details, outcome measures, and results. Extracted data were synthesized narratively.

## 3. Mechanisms of action

### 3.1. Neurobiological mechanism

**Figure 2.**
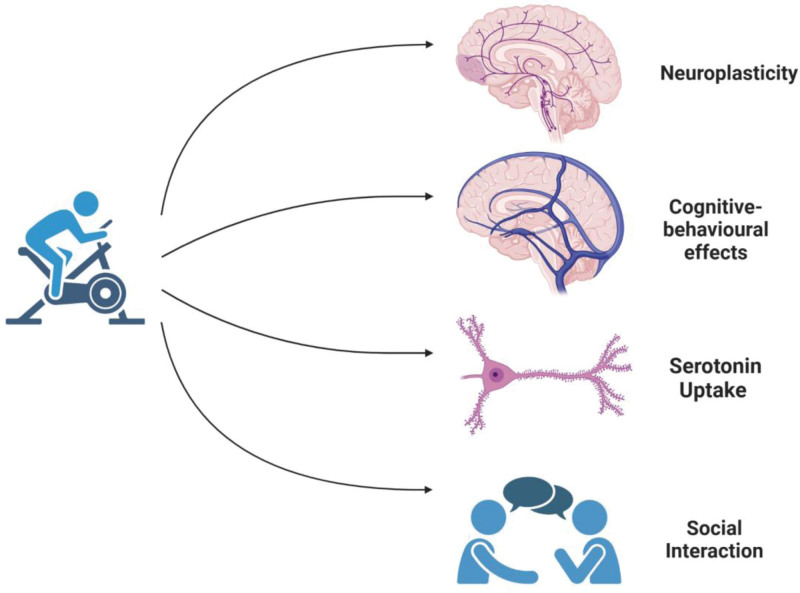
Aerobic exercise role in generalized anxiety disorder.

Regular aerobic exercise has been shown to increase the availability of tryptophan in the brain by enhancing its uptake across the blood-brain barrier (Fig. 2).^[17]^ This increased availability of tryptophan facilitates the synthesis of serotonin, leading to elevated levels of this crucial neurotransmitter in the brain.^[18]^ Research indicates that aerobic exercise not only boosts the concentration of serotonin but also promotes the release of serotonin from presynaptic neurons into the synaptic cleft, where it can bind to serotonin receptors on postsynaptic neurons.^[19]^ The activation of these receptors initiates a series of intracellular signaling cascades that modulate neuronal excitability and synaptic transmission, ultimately influencing mood and anxiety levels.^[17]^ Additionally, aerobic exercise enhances serotonin signaling by upregulating the expression of specific serotonin receptors, particularly the 5-HT1A receptor, which is integral to mediating the anxiolytic effects of serotonin.^[20]^

Moreover, the effects of aerobic exercise on serotonin dynamics are likely influenced by the intensity and duration of the activity.^[5]^ Higher intensity workouts may elicit more significant increases in serotonin levels compared to moderate-intensity exercises.^[6]^ Investigating these nuances can provide deeper insights into optimizing exercise prescriptions for anxiety reduction. Aerobic exercise also promotes neuroplasticity,^[21]^ a process essential for the brain’s adaptation to stressors and changes in the environment. Through mechanisms such as increased neurogenesis and synaptogenesis, exercise induces structural changes in the brain, particularly in regions critical for emotional regulation and stress response, such as the hippocampus and prefrontal cortex.^[22]^ The generation of new neurons in these areas enhances cognitive and emotional processing, allowing for improved resilience against anxiety.^[23]^

The molecular mechanisms underlying the neuroplastic effects of aerobic exercise are complex and multifaceted. A key mediator of exercise-induced neuroplasticity is BDNF.^[24]^ Aerobic exercise upregulates BDNF expression in the brain, especially in regions such as the hippocampus and prefrontal cortex, where it plays a pivotal role in promoting neurogenesis and synaptogenesis.^[24]^ BDNF not only supports the survival and differentiation of neurons but also enhances synaptic plasticity by modulating the activity of neurotransmitter systems, including glutamate and gamma-aminobutyric acid (GABA), which are critical for synaptic transmission and plasticity.^[25]^ Furthermore, recent studies have indicated that different types and intensities of aerobic exercise can differentially influence BDNF levels. High-intensity interval training (HIIT) leads to more pronounced increases in BDNF compared to steady-state aerobic activities, suggesting that tailored exercise interventions could optimize neuroplastic benefits and support anxiety management.^[12,25]^ These neuroplastic changes have been associated with significant improvements in mood, cognitive function, and resilience to stress, indicating that the relationship between aerobic exercise and anxiety is underpinned by both biochemical and structural brain changes.^[22]^

### 3.2. Psychological mechanisms

Engaging in regular aerobic exercise has profound cognitive-behavioral effects on anxiety regulation.^[26]^ One of the primary psychological benefits of exercise is its ability to distract individuals from negative thoughts and worries, redirecting attention toward physical sensations and the present moment.^[27]^ This mindfulness aspect of exercise can help break the cycle of rumination often associated with anxiety, allowing individuals to focus on their bodies and movements instead of their anxieties. Moreover, aerobic exercise provides opportunities for mastery and accomplishment, which can significantly enhance self-efficacy – the belief in one’s ability to succeed in specific situations.^[28]^ The sense of achievement that comes from setting and reaching fitness goals can foster greater confidence in coping with stressors outside of the exercise environment. This can lead to a broader application of resilience skills in daily life, as individuals learn to tackle challenges with a more positive mindset. In addition, aerobic exercise acts as a natural stress reliever by stimulating the release of endorphins and other neurochemicals, such as norepinephrine and dopamine that promote relaxation and alleviate tension.^[28]^ This biochemical response not only contributes to an immediate sense of well-being but also plays a role in long-term emotional regulation.

Regular exercise also provides a structured outlet for pent-up energy and emotions, allowing individuals to channel stress constructively rather than letting it accumulate. This physical release can be particularly beneficial for those who struggle with expressing their emotions verbally or managing stress through traditional coping mechanisms.^[29]^ Over time, consistent engagement in physical activity enables individuals to learn and internalize effective coping strategies, such as deep breathing, progressive muscle relaxation, and mindfulness techniques. These strategies can then be applied in various contexts to manage anxiety and promote emotional well-being.^[26]^

Participation in group-based aerobic exercise programs further amplifies these psychological benefits by offering opportunities for social support and interaction.^[30]^ The camaraderie and shared experiences fostered within exercise groups provide emotional support, encouragement, and motivation, which are crucial for enhancing adherence to exercise regimens and promoting overall psychological well-being.^[31]^ Moreover, group exercise settings facilitate social engagement, connection, and a sense of belonging, which are essential for combating feelings of isolation and loneliness often associated with anxiety disorders.^[32]^ Social interaction itself has been shown to have beneficial effects on mood and anxiety. Engaging in conversations, laughter, and shared activities during exercise sessions can elevate mood, alleviate stress, and foster feelings of connectedness with others.^[33]^

The positive social experiences derived from group exercise serve as buffers against the negative effects of anxiety, creating a supportive environment where individuals can challenge fears, build confidence, and experience a sense of belonging.^[33]^ Additionally, the group dynamic can help normalize the experience of anxiety, allowing individuals to realize they are not alone in their struggles. Future research could further explore the nuances of these group dynamics, examining how different types of group exercise (e.g., team sports vs fitness classes) uniquely contribute to psychological resilience and anxiety management.

## 4. Current evidence from existing literature

The efficacy of exercise interventions in alleviating symptoms of anxiety disorders has been investigated across several studies (Table 1). LeBouthillier and Asmundson (2017)^[34]^ conducted an RCT comparing the efficacy of aerobic exercise and resistance training in individuals with anxiety-related disorders. Their findings revealed that both forms of exercise were effective in improving disorder status. Specifically, aerobic exercise led to reductions in general psychological distress and anxiety symptoms, while resistance training targeted disorder-specific symptoms, anxiety sensitivity, distress tolerance, and intolerance of uncertainty. Moreover, the study found that physical fitness played a role in predicting reductions in distress. Similarly, Henriksson et al (2022)^[35]^ conducted an RCT to examine the effects of exercise interventions on anxiety symptoms in primary care patients. Their study demonstrated that both low-intensity and moderate/high-intensity exercise interventions resulted in significant improvements in anxiety and depressive symptoms compared to a control group. These findings suggest that exercise, regardless of intensity, can be beneficial in reducing symptoms of anxiety disorders.

**Table 1:** Study characteristics.

Author and year	Study design	Sample size	Duration	Positive outcomes of exercise on anxiety	Adverse effects
LeBouthillier and Asmundson (2017)^[34]^	RCT	48	4 wk	Improvement in disorder status, general psychological distress, anxiety sensitivity, distress tolerance, and intolerance of uncertainty.	Not reported
Henriksson et al (2022)^[35]^	RCT	286	12 wk	Reduction in anxiety and depressive symptoms.	Not reported
Vancini et al (2017)^[36]^	RCT	63	8 wk	Improvement in depression, anxiety, and quality of life.	Not reported
Blumenthal et al (2021)^[37]^	RCT	128	12 wk	Reduction in anxiety symptoms.	Not reported
Loh et al (2019)^[38]^	RCT	252	6 wk	Improvement in anxiety, mood, social, and emotional well-being.	Not reported
Gordon et al (2020)^[39]^	RCT	28	8 wk	Reduction in anxiety symptoms.	Not reported
Gordon et al (2021)^[40]^	RCT	44	8 wk	Improvement in anxiety symptoms.	Not reported
Plag et al (2020)^[41]^	RCT	33	12 d	Moderate to large effects on anxiety and stress-related bodily symptoms.	Not reported
Ma et al (2017)^[42]^	RCT	86	3 mo	Improvement in anxiety levels and metabolic functions.	Not reported
Jazaieri et al (2012)^[43]^	RCT	56	Not specified	Reduction in social anxiety and depression.	Not reported
Oeland et al (2010)^[44]^	Non-blinded controlled study	48	20 wk	Increase in physical activity and VO2max.	Not reported

Furthermore, Vancini et al (2017)^[36]^ compared the effects of Pilates and walking on quality of life, depression, and anxiety levels in overweight and obese individuals. Both Pilates and walking groups demonstrated improvements in quality of life, depression, and trait-anxiety levels, with state-anxiety levels improving only in the walking group. In addition, Blumenthal et al (2021)^[37]^ conducted a randomized clinical trial to determine the effects of exercise and escitalopram on anxiety symptoms in patients with coronary heart disease. Both exercise and escitalopram groups showed greater reductions in anxiety symptoms compared to the placebo group, with escitalopram resulting in less anxiety compared to exercise. Participants randomized to the exercise group reported greater reductions in HADS-A (exercise, −4.0; 95% CI: −4.7 to −3.2) compared with those randomized to placebo (−3.5; 95% CI: −4.5 to −2.4; *P* = .03); participants randomized to escitalopram reported less anxiety compared with those randomized to exercise (−1.67; 95% CI: −2.68 to −0.66; *P* = .002).

Loh et al (2019)^[38]^ investigated the effects of a home-based exercise program on anxiety and mood disturbances in older adults with cancer receiving chemotherapy. The exercise group showed significantly improved anxiety, mood, and social and emotional well-being compared to the control group. Moreover, Gordon et al (2020,^[39]^ 2021^[40]^) conducted 2 RCTs evaluating the effects of resistance exercise training (RET) on anxiety and worry symptoms among young adults and those with subclinical generalized anxiety disorder (GAD). Both studies demonstrated significant reductions in anxiety and worry symptoms following RET interventions. RET significantly reduced anxiety symptoms from baseline to post-intervention (mean difference = −7.89, *P* ≤ .001).^[39]^

Plag et al (2020)^[41]^ specifically targeted individuals diagnosed with GAD to assess the effectiveness of HIIT as an intervention. HIIT involves short bursts of intense exercise followed by periods of rest or lower-intensity activity. Their study revealed that participants who underwent HIIT experienced significant reductions in symptoms of anxiety and any related conditions when compared to individuals in a lower-intensity training group. This suggests that the intensity of the exercise regimen plays a crucial role in mitigating anxiety symptoms and associated comorbidities in individuals with GAD.

Ma et al (2017)^[42]^ conducted a study to assess the impact of a home-based exercise program on anxiety levels and metabolic functions in individuals diagnosed with anxiety disorders in Taiwan. Their findings indicated that the exercise program led to improvements in anxiety levels and metabolic indicators and even reduced the prevalence of metabolic syndrome. In a separate study, Jazaieri et al (2012)^[43]^ compared mindfulness-based stress reduction to aerobic exercise as an intervention for social anxiety disorder (SAD). Both interventions yielded positive outcomes, with participants experiencing reductions in social anxiety and depression, along with increases in subjective well-being. Oeland et al (2010)^[44]^ delved into the effects of exercise on individuals grappling with depression and anxiety. Their research revealed that participants in the exercise intervention group demonstrated enhanced physical activity levels and improved physical fitness compared to those in the control group.

## 5. Implications for practice and future directions

The reviewed literature demonstrates a consistent pattern of evidence supporting the efficacy of exercise interventions in alleviating symptoms of anxiety disorders. Studies included in the review encompassed various exercise modalities, including aerobic exercise, resistance training, HIIT, and mindfulness-based interventions. Across these diverse approaches, significant improvements in anxiety symptoms, psychological distress, and overall well-being were observed among participants with anxiety-related disorders.

Comparing the findings across studies reveals several common themes and trends. Aerobic exercise and resistance training emerged as effective interventions for reducing anxiety symptoms and improving psychological well-being. While aerobic exercise primarily targeted general psychological distress and anxiety symptoms, resistance training showed promise in addressing disorder-specific symptoms and enhancing distress tolerance. Additionally, studies investigating different exercise intensities, such as low-intensity and moderate/high-intensity exercise, consistently demonstrated significant improvements in anxiety and depressive symptoms compared to control groups, indicating that exercise intensity may not be a decisive factor in its therapeutic benefits. Moreover, interventions targeting specific populations, such as individuals with GAD or SAD, yielded positive outcomes, further underscoring the broad applicability of exercise interventions across diverse populations with anxiety disorders.

The findings significantly affect theory, research, practice, and policy. From a theoretical perspective, the evidence supports the role of exercise as a non-pharmacological intervention for managing anxiety symptoms, complementing existing theoretical frameworks of anxiety regulation and stress management. In terms of practice, the incorporation of exercise interventions into clinical treatment plans for anxiety disorders is warranted, considering their demonstrated efficacy and favorable side effect profiles. Additionally, public health initiatives promoting physical activity for mental health benefits should prioritize addressing anxiety disorders, given their high prevalence and substantial impact on individual well-being and societal costs. From a policy standpoint, the integration of exercise prescriptions into healthcare guidelines and the development of community-based programs targeting adults with anxiety disorders are recommended to enhance access to evidence-based interventions and promote population-wide mental health.

There is a pressing need for research on the practical implementation of exercise interventions in real-world settings, such as primary care, community programs, and digital platforms. Future studies should investigate the barriers and facilitators to exercise adherence specifically in individuals with GAD and explore strategies for integrating exercise prescriptions into standard clinical care. Also, longitudinal studies with larger sample sizes and diverse populations are needed to establish the long-term efficacy and sustainability of exercise interventions for anxiety disorders. Comparative effectiveness studies evaluating different exercise modalities, intensities, and delivery formats can inform personalized treatment approaches tailored to individual needs and preferences. Mechanistic studies investigating the underlying neurobiological, psychological, and social mechanisms through which exercise exerts its therapeutic effects are essential for elucidating the action pathways and optimizing intervention strategies. Additionally, research examining the implementation and scalability of exercise interventions in real-world settings, including healthcare systems, community organizations, and digital platforms, can inform policy decisions and public health initiatives to promote mental well-being through physical activity.

## 6. Limitations of review

Despite the overall positive findings, several limitations should be acknowledged. The heterogeneity among study designs, participant characteristics, and outcome measures complicates direct comparisons and generalizability of findings. Methodological limitations, such as small sample sizes, short follow-up periods, and lack of long-term outcomes, restrict the robustness and reliability of the evidence. Moreover, publication bias and selective reporting may influence the interpretation of results, potentially overestimating the effectiveness of exercise interventions.

## 7. Conclusion

The literature reviewed provides compelling evidence supporting the efficacy of exercise interventions in alleviating symptoms of anxiety disorders. Across various exercise modalities, including aerobic exercise, resistance training, HIIT, and mindfulness-based interventions, significant improvements in anxiety symptoms, psychological distress, and overall well-being were consistently observed among individuals with anxiety-related disorders. These findings underscore the potential of exercise as a non-pharmacological intervention for managing anxiety and promoting mental health. While the evidence is promising, several considerations should be taken into account. Methodological limitations, such as heterogeneity among study designs, participant characteristics, and outcome measures, highlight the need for robust research methodologies and standardization of intervention protocols. Moreover, addressing publication bias, selective reporting, and methodological biases is crucial for ensuring the reliability and validity of findings. Moving forward, future research should focus on longitudinal studies with larger and more diverse populations to establish the long-term efficacy and sustainability of exercise interventions for anxiety disorders. Comparative effectiveness studies evaluating different exercise modalities and delivery formats can inform personalized treatment approaches tailored to individual needs. Mechanistic research exploring the underlying neurobiological, psychological, and social mechanisms of exercise’s therapeutic effects is essential for optimizing intervention strategies and elucidating action pathways. Integrating exercise prescriptions into clinical treatment plans and public health initiatives can enhance access to evidence-based interventions and promote population-wide mental well-being.

## Author contributions

**Conceptualization:** Zimakor Ewuzie.

**Writing – original draft:** Zimakor Ewuzie, Chimezirim Ezeano, Nicholas Aderinto.

**Writing – review & editing:** Zimakor Ewuzie, Chimezirim Ezeano, Nicholas Aderinto.
